# Improving the Oxidation Resistance of Phenolic Resin Pyrolytic Carbons by In Situ Catalytic Formation of Carbon Nanofibers via Copper Nitrate

**DOI:** 10.3390/ma17153770

**Published:** 2024-07-31

**Authors:** Zhi Wu, Pengcheng Jiang, Hongxing Pang, Guanghai Cheng, Jiajun Li, Hao Liu, Yan Ma, Yunjie Dong, Zhoufu Wang

**Affiliations:** 1 School of Materials Science and Engineering, Hunan Institute of Technology, Hengyang 421002, China; wuzhi0549@163.com (Z.W.); jiangpengchengcn@163.com (H.P.); 15737620632@163.com (G.C.); 18374837648@163.com (J.L.); 2 The State Key Laboratory of Refractories and Metallurgy, Wuhan University of Science & Technology, Wuhan 430081, China; wustlh@163.com (H.L.); yanmacn@163.com (Y.M.); jdzthd2016@163.com (Y.D.)

**Keywords:** phenolic resin, copper nitrate, carbon nanofibers, graphitization, oxidation resistance

## Abstract

Phenolic resin pyrolytic carbons were obtained by catalytic pyrolysis of phenolic resin at 500 °C, 600 °C, 700 °C, and 800 °C for 3 h in an argon atmosphere using copper nitrate as a catalyst precursor. The effects of copper salts on the pyrolysis process of phenolic resin as well as the structural evolution and oxidation resistance of phenolic resin pyrolytic carbons were studied. The results showed that copper oxide (CuO) generated from the thermal decomposition of copper nitrate was reduced to copper (Cu) by the gas generated from the thermal decomposition of the phenolic resin. Carbon nanofibers with tapered structures were synthesized by Cu catalysis of pyrolysis gas at 500–800 °C. The catalytic pyrolysis of phenolic resin with Cu increased the graphitization degree and reduced the pore volume of the phenolic resin pyrolytic carbons. The combined action improved the oxidation resistance of phenolic resin pyrolytic carbons.

## 1. Introduction

Phenolic resins are widely utilized as binders in carbon composite materials due to their suitable viscosity, excellent wetting properties, and high char yield [1]. During the thermal decomposition of phenolic resin, a three-dimensional cross-linked network is established via curing cross-linking reactions, enhancing the material’s strength through the stabilization of the bonding interface [2,3,4]. However, the pyrolysis process of phenolic resin occurs between 200 °C and 750 °C, and small molecules such as H_2_O, CO, CO_2_, and H_2_ are derived from different structure units of the phenolic resin crosslinking during different heat treatments in the pyrolysis process [5]. The formation of pores in the material due to the pyrolysis of phenolic resin results in a decrease in material strength. The isotropic glass carbons derived from phenolic resin pyrolysis exhibit challenges in achieving graphitization, leading to inferior oxidation resistance of the materials [6,7,8].

Transition metals (Fe, Co, Ni) have been used to catalyze phenolic resin to generate carbon nanotubes (CNTs) or carbon nanofibers (CNFs) to improve their performance [9,10,11]. Hu et al. used nickel oxalate to catalyze the graphitization of phenolic resin pyrolytic carbons and generated CNFs at 1000 °C [12]. Wang et al. synthesized CNTs via catalyzed pyrolysis of phenolic resin at 800 °C with ferric nitrate. The synthesis temperatures of CNTs or CNFs catalyzed by transition metals are usually 800 °C and above [13]. However, the phenolic resin releases molecule gases and forms an isotropic pyrolytic carbon structure during the pyrolysis at a temperature below 800 °C. Materials for the catalytic modification of phenolic resin should operate at low temperatures.

The research on the catalytic formation of carbon nanofibers at low temperatures from gaseous carbon sources using metal Cu via chemical vapor deposition (CVD) has received widespread attention in recent years. Nano-sized Cu particles, serving as catalysts, have the capability to significantly lower the temperature necessary for carbon deposition, thereby obviating the requirement for growth promoters in catalyzing gaseous carbon sources [14,15,16]. Shaikjee et al. prepared CNFs by a chemical vapor deposition method at 250 °C using acetylene as a carbon source and copper nitrate as a catalyst precursor [17]. Phenolic resin produces excessive small molecules of gas such as CH_4_, H_2_, and CO during pyrolysis, and Cu may catalyze the pyrolytic gas into CNFs or CNTs. However, the structure and properties of Cu-catalyzed phenolic resin have not yet been reported [18]. Therefore, it is important to investigate the effects of copper salts on the pyrolysis process of phenolic resin as well as the structure and properties of phenolic resin pyrolytic carbons at low temperatures.

Here, phenolic resin was the raw material, and copper nitrate was the catalyst precursor. Phenolic resin with different amounts of copper nitrate was pyrolyzed at 500–800 °C for 3 h in an argon atmosphere. The effects of copper salts on the pyrolysis process of phenolic resin as well as the structural evolution and oxidation resistance of phenolic resin pyrolytic carbons were studied. The mechanism by which Cu catalyzes the synthesis of CNFs at low temperatures was explored.

## 2. Materials and Methods

### 2.1. Raw Materials

Thermosetting phenolic resin (viscosity = 16–19 Pa·s, solid content = 77–83%, free phenol = 10.3–12.5%, carbon yield ≥ 56%) was the raw material and was supplied by Shengquan (Jinan, China). Copper nitrate (Cu(NO_3_)_2_·3H_2_O, AR, ≥99%) was the catalyst precursor and was provided by Aladdin (Shanghai, China). Ethyl alcohol (C_2_H_5_OH, AR, ≥99%) was the solvent and was provided by Guoyao (Shanghai, China).

### 2.2. Sample Preparation

Copper nitrate at a mass ratio of 0.5 wt%, 1.0 wt%, 2.0 wt%, and 3.0 wt% (copper nitrate to phenolic resin) was dissolved in ethyl alcohol (20 mL) and stirred evenly; the mixed solution was then slowly added to liquid phenolic resin and stirred well. The mixture was then cured at 80 °C for 12 h, at 110 °C for 12 h, and at 180 °C for 24 h. The cured samples with 0.5 wt%, 1.0 wt%, 2.0 wt%, and 3.0 wt% copper nitrate were designated as PF0.5, PF1.0, PF2.0, and PF3.0, respectively. The cured phenolic resin without catalyst was designated as PF. Finally, the cured samples were placed in a tube furnace under an argon atmosphere and heated to 300 °C for 1 h at a rate of 5 °C/min and then heated to 500 °C, 600 °C, 700 °C, and 800 °C for 3 h at a rate of 3 °C/min. The phenolic resin pyrolytic carbons were thus obtained.

### 2.3. Characterization

The phenolic resin pyrolysis and oxidation resistance of phenolic resin pyrolytic carbons were identified by using a thermogravimetric–differential scanning calorimeter (TG-DSC, STA 449C, Netzsch, Selb, Germany). The oxidation activation energy was calculated based on the Arrhenius Equation (1) as follows [19]:

(1) $${K=A_{a}e}^{\frac{-E_{a}}{RT}}$$

where *T* is the reaction temperature, *K* is the oxidation reaction rate at temperature *T*, *R* is the gas constant, *E_a_* is the apparent activation energy of the oxidation reaction, and *A_a_* is the apparent frequency factor in the oxidation reaction. The oxidation reaction kinetic Equation (2) of phenolic resin pyrolytic carbons was obtained by taking logarithms of the Arrhenius equation as follows [20]:

(2) $$\mathrm{Ln}\left(T\frac{\mathrm{dW}}{\mathrm{dT}}\right)=\ln\left(A_{a}\right)\;-\;\frac{E_{a}}{R}\times \frac{1}{T}$$

The activation energy (E_a_) and frequency factor (A_a_) of the oxidation reactions are obtained from the slopes and intercepts of the $\ln\left(T\frac{\mathrm{dW}}{\mathrm{dT}}\right)$ and 1/T plots, respectively.

The crystalline structure and graphitization degree of pyrolyzed resin were analyzed by X-ray diffraction (XRD, Philips X’pert Pro, PANalytical, Almelo, The Netherlands) and Raman spectra (Renishaw/inVia Qontor, Bruker, Billerica, MA, USA), respectively. The microstructure of pyrolyzed resin was observed via scanning electron microscopy (SEM, ISM-6610, JEOL, Tokyo, Japan) and transmission electron microscopy (TEM, 2100 UHR STEM/EDS, JEOL, Tokyo, Japan) with energy-dispersive spectroscopy (EDS, INCA IE 350 PentaFET X-3, Oxford, UK). The pore structure of pyrolyzed resin was analyzed by Brunauer–Emmett–Teller (BET, ASAP 2020, Micromeritics, Norcross, GA, USA).

## 3. Results and Discussion

### 3.1. Pyrolysis Process of Phenolic Resin

**Figure 1:**
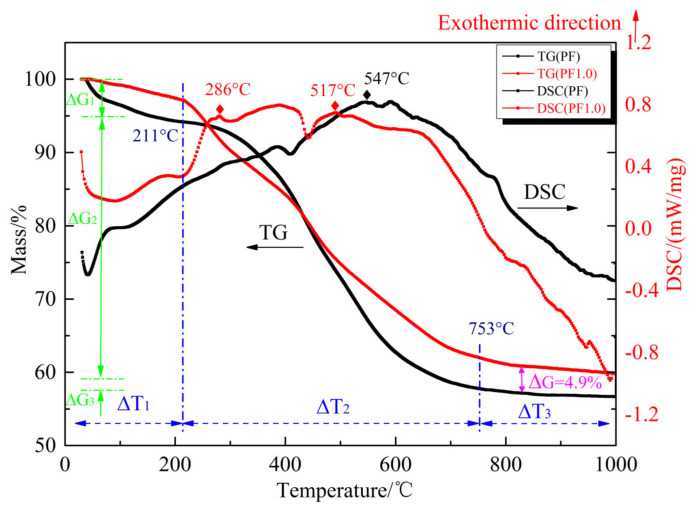
TG–DSC curves of sample PF and sample PF1.0.

The samples PF and PF1.0 were heated in an argon atmosphere at the rate of 10 °C/min from 30 °C to 1000 °C, and the resulting TG–DSC curves were obtained as shown in Figure 1.

The TG curves show that there were three stages of the pyrolysis weight loss of PF and PF1.0. The first stage of pyrolysis weight loss was 30–211 °C, where the phenolic resin loses weight slightly (ΔG_1_ = 5%). The resulting pores of cured phenolic resin could absorb a small amount of water in the air; the weight loss of phenolic resin in the first stage was due to the adsorbed water volatilized during pyrolysis. The dehydration condensation reaction of phenolic resin also resulted in weightlessness. The second stage of pyrolysis weight loss was 211–753 °C, where the phenolic resin loses weight rapidly (ΔG_2_ = 40%). Small molecular gases such as CH_4_, H_2_, and CO were released due to the breaking of methylene bonds and ether bonds in the phenolic resin, thus resulting in rapid pyrolysis weight loss. The mass loss of sample PF1.0 was lower than that of sample PF, indicating that the addition of a catalyst could delay the pyrolysis weight loss of the phenolic resin. The third stage of pyrolysis weight loss (ΔG_3_ = 3%) was 753–1000 °C. The pyrolysis weight loss of phenolic resin was due to the aromatic ring dehydrogenation reaction. The carbon yield of sample PF1.0 was 4.9% higher than that of sample PF, indicating that the introduction of copper salts could improve the carbon yield of the phenolic resin pyrolytic carbon. The DSC curves of PF1.0 had a relative exothermic peak at 286 °C because the CuO decomposed from copper nitrate was reduced to Cu by CH_4_ and CO gases with the release of heat [21]. The DSC curves show that the temperature of the maximum exothermic peak of the phenolic resin increased from 547 °C to 517 °C upon introduction of copper salts.

### 3.2. Crystal Structure of Phenolic Resin Pyrolytic Carbons

XRD and Raman spectroscopy were used to analyze the phase compositions and graphitization degree of the phenolic resin pyrolytic carbons, respectively. The effects of copper salts and pyrolysis temperature on the crystal structure of phenolic resin pyrolytic carbons were investigated.

**Figure 2:**
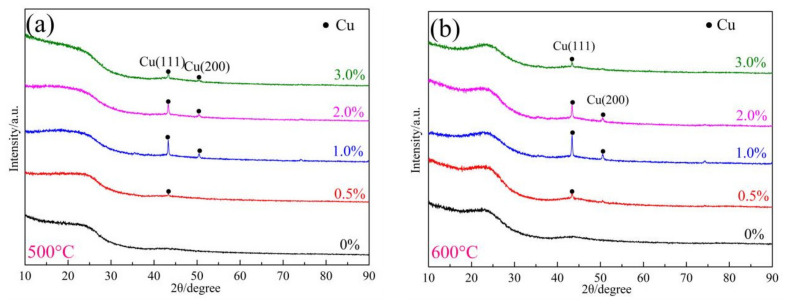

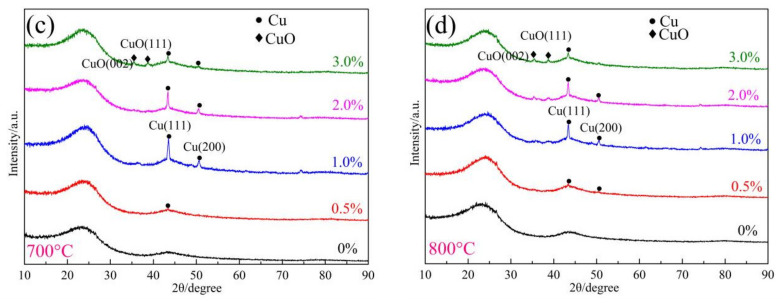
The XRD patterns of samples obtained at 500–800 °C: (**a**) 500 °C; (**b**) 600 °C; (**c**) 700 °C; and (**d**) 800 °C.

Figure 2 displays the XRD patterns of phenolic resins with varying amounts of copper nitrate (0 wt%, 0.5 wt%, 1.0 wt%, 2.0 wt%, and 3.0 wt%) after undergoing heat treatment at temperatures ranging from 500 to 800 °C for 3 h. At 500 °C and 600 °C, the XRD patterns in Figure 2a,b only show the diffraction peak of Cu (ICDD 03-065-9026) without any peak of CuO. This suggests that the CuO formed from the thermal decomposition of copper nitrate in the sample is reduced to Cu by the reducing gas produced during the pyrolysis of phenolic resin. This phenomenon is primarily attributed to the rapid pyrolysis of phenolic resin and the release of a significant amount of reducing gas at these temperatures. As the heat treatment temperature is raised to 700 °C and 800 °C, the samples depicted in Figure 2c,d display Cu diffraction peaks close to the diffraction angles of 43.3° and 50.5°. Furthermore, in sample PF3.0, faint diffraction peaks emerge near the diffraction angles of 35.5° and 38.5°, which correspond to the diffraction peaks of the (002) and (111) crystal planes of CuO (ICCD 01-089-2530). This indicates that an excessive amount of copper nitrate results in weaker diffraction peaks of the crystal planes of CuO. At this temperature, the pyrolysis gas amount of phenolic resin is insufficient to reduce all CuO to elemental Cu. The results show that when copper particles are introduced in the form of CuO, the addition amount and heat treatment temperature both affect the phase composition of phenolic resin pyrolytic carbon. When the heat treatment temperature is 500 °C and 600 °C, the reductive gas generated by the pyrolysis of phenolic resin is sufficient, and the CuO with different addition amounts can be reduced to elemental Cu. However, when the amount of copper nitrate is too much (3.0 wt%) and the heat treatment temperature is high (700 °C and 800 °C), the gas generated by the pyrolysis of phenolic resin is not enough to fully reduce all CuO to Cu, which may affect the catalytic graphitization effect of resin pyrolysis carbon. The diffraction peak of Cu is the sharpest in the samples with 1.0 wt% Cu nitrate, indicating that this amount of Cu nitrate addition is more suitable [22].

**Figure 3:**
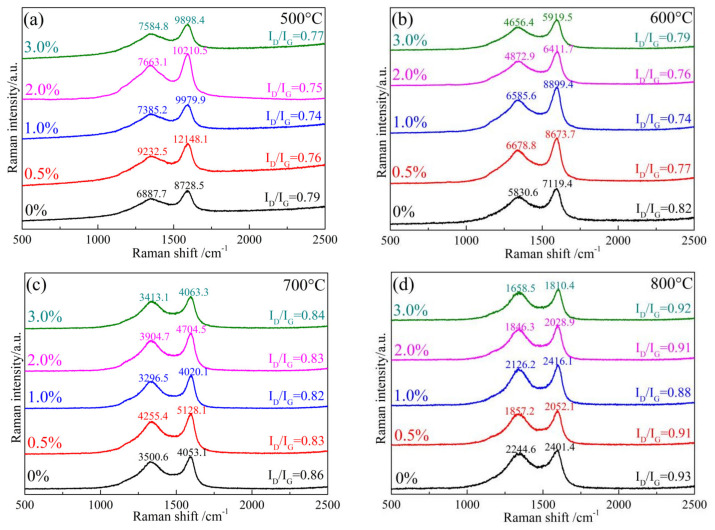
The Raman spectra of samples obtained at 500–800 °C: (**a**) 500 °C; (**b**) 600 °C; (**c**) 700 °C; and (**d**) 800 °C.

Figure 3 displays the Raman spectra of samples containing varying amounts of copper nitrate after undergoing pyrolysis at temperatures ranging from 500 °C to 800 °C for a duration of 3 h.

Raman analysis shows two characteristic bands at 1344 cm^−1^ (D band) and 1604 cm^−1^ (G band) for the phenolic resin pyrolytic carbon. The D band at 1344 cm^−1^ corresponds to the defects, vacancies, and glassy carbons of phenolic resin pyrotic carbons; the G band at 1604 cm^−1^ corresponds to the carbon stretching in SP^2^ of the graphite layer [23]. The R value (R = I_D_/I_G_) is the intensity of the D band to G band and was used to monitor the graphitization of phenolic resin pyrolytic carbons. A high R value indicates a smaller degree of graphitization of phenolic resin pyrolytic carbons [24].

The results show that the R value of the samples with copper nitrate at varying temperatures consistently exhibits a lower value compared to the samples without copper nitrate. This suggests that the degree of graphitization of the phenolic resin pyrolytic carbon can be enhanced when CuO is reduced to Cu. Furthermore, the amounts of copper nitrate also impact the graphitization degree of phenolic resin pyrocarbon. As the additive amount increased from 0.5 wt% to 3.0 wt%, the R value of the samples initially decreased and then rose again. Notably, the sample PF1.0 exhibited the lowest R value, indicating that the graphitization degree of the sample pyrocarbon corresponding to the addition amount of copper nitrate at 1.0 wt% is the highest. The results show that when Cu particles are introduced with CuO, the effect of Cu particles on graphitization degree of phenolic resin pyrolytic carbon is the same as that when Cu particles are directly introduced. Compared with the direct introduction of elemental Cu, the reduction of elemental Cu from CuO can significantly improve the graphitization degree of phenolic resin pyrolysis carbon at 500 °C and 600 °C. This is due to the increased presence of pyrolysis gases from the resin at these temperatures, leading to a more pronounced reduction effect.

### 3.3. Microstructure of Phenolic Resin Pyrolytic Carbons

The crystal structure of phenolic resin pyrolytic carbons was affected by the pyrolysis temperature and copper salt. The corresponding changes in the microstructure were analyzed by SEM and TEM.

**Figure 4:**
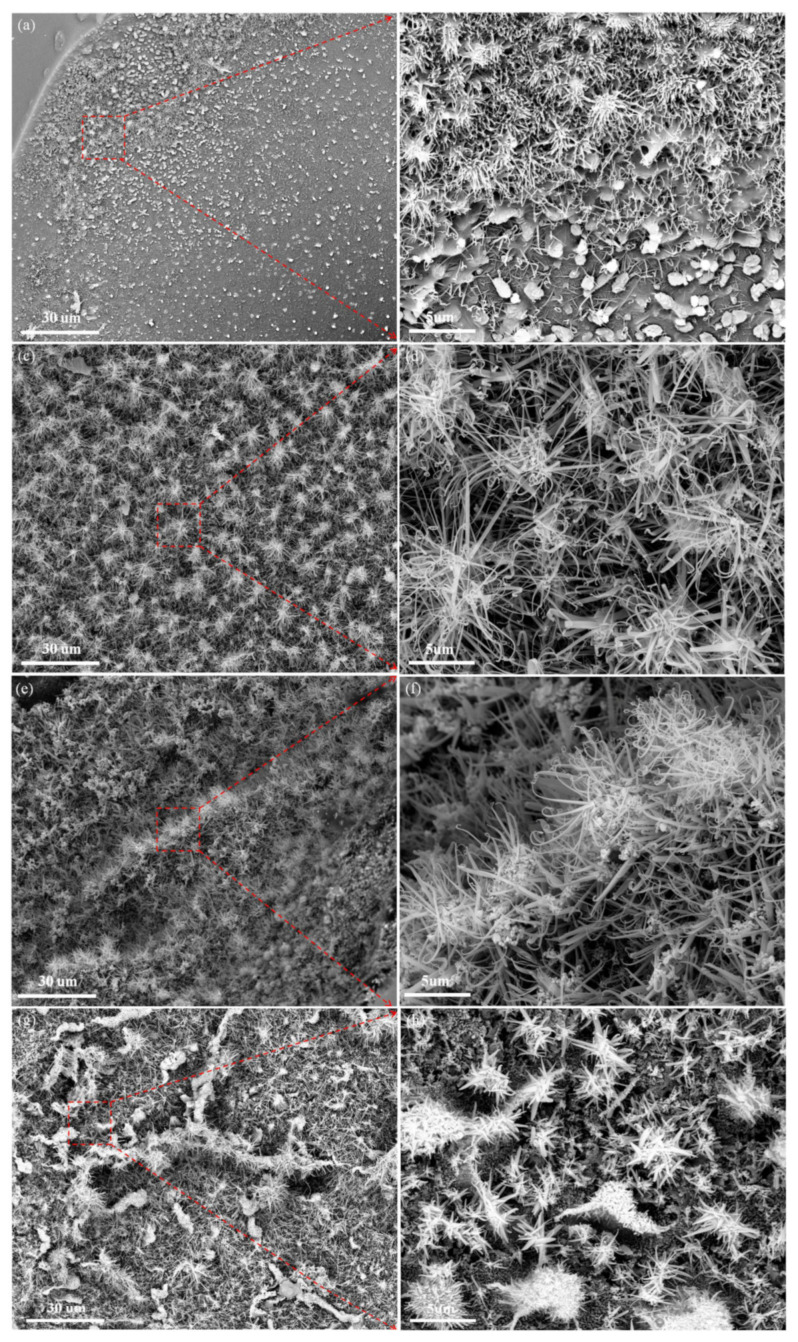
SEM images of samples PF0.5 (**a**,**b**), PF1.0 (**c**,**d**), PF2.0 (**e**,**f**), and PF3.0 (**g**,**h**) heated at 500 °C for 3 h. (The image on the right is an enlarged view of the red squares).

Figure 4 shows the CNFs with tapered structures were found in the phenolic resin pyrolytic carbons with the catalyst added. These materials were tightly packed to form a clustered structure.

**Figure 5:**
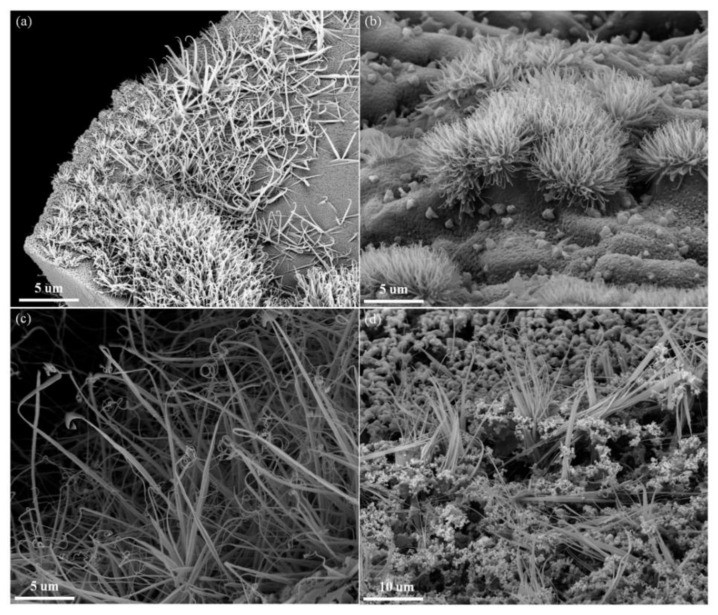
SEM images of sample PF0.5 heated at various temperatures for 3 h: (**a**) 500 °C; (**b**) 600 °C; (**c**) 700 °C; and (**d**) 800 °C.

Many CNFs were generated on the surface of the pyrolyzed resin matrix (Figure 4a) when the catalyst content was 0.5 wt%. Figure 4b is a partial enlargement of Figure 4a (the red square). Figure 5b shows the CNF tip had a small amplitude of curl, and the length of the CNFs was less than 5 µm. Figure 4c shows that the amount of CNFs in the pyrolyzed resin matrix was significantly increased when the catalyst content increased to 1.0 wt%. The CNFs were tightly packed with each other, forming a clustered structure. Figure 4d shows that the length of the CNFs was less than 10 µm, and the diameter of the CNFs ranged from tens to hundreds of nanometers. The matrix of the phenolic resin pyrolytic carbons was covered with CNFs when the catalyst content was 2 wt% (Figure 4e). Figure 4f further shows that the length of CNFs was still less than 10 µm, and a small number of nanoparticles were observed at the bottom of the cluster structure. Figure 4g shows that the number of CNFs in the phenolic resin pyrolytic carbons decreased significantly with 3.0 wt% catalyst. Figure 4h shows that the nanoparticles were obviously agglomerated with only a small amount of CNFs in the clustered structure, indicating poor dispersion of the catalyst and limited its catalytic activity.

Figure 5 shows the CNFs were densely packed to form the clustered structure. The length of the CNFs was less than 5 μm at 500 °C (Figure 5a). The CNFs continued to grow, and the length of the CNFs was 5–10 μm at 600 °C (Figure 5b). The resulting clustered structure was tightly packed. The length of CNFs reached 30 μm at 700 °C (Figure 5c), indicating that increasing heat treatment temperature could further promote the growth and development of CNFs, but the numbers of CNFs were decreased. At 800 °C, the number of CNFs decreased (Figure 5d) due to reduced pyrolytic gas in the phenolic resin at 800 °C. This lack of pyrolysis gas as the carbon source limited the catalytic performance of Cu.

This result suggests that the catalyst content and heat treatment temperature affected the growth of the CNFs. The number of CNFs was affected by the content of catalyst, and an increase in the content of catalyst could in turn increase the number of CNFs produced; excessive catalyst addition would affect the catalyst dispersion and limit its catalytic activity. The growth and development of CNFs were affected by the heat treatment temperature; increased heat treatment temperature would increase the length of the CNFs but reduce the number of CNFs.

**Figure 6:**
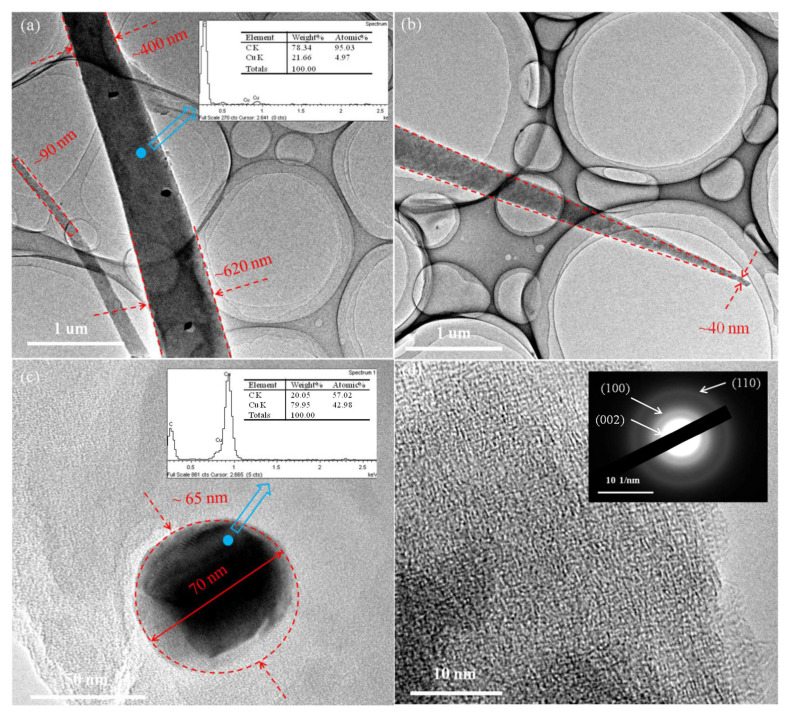
TEM images of sample PF1.0 heated at 500 °C for 3 h: (**a**) The bottom of carbon nanofiber (The spectrum corresponds to the blue circle in the diagram); (**b**) The cusp of carbon nanofiber; (**c**) carbon nanofiber with catalyst particle (The spectrum corresponds to the blue circle in the diagram); (**d**) HR-TEM of carbon nanofiber.

Figure 6a shows the bottom structure of the resulting nanofibers. The EDS spectra of the blue region contained C and Cu elements, which further proved that the generated tapered structure included CNFs.

The diameter of the CNFs gradually decreased along the growth direction, and the diameter at the base of the CNFs was 620 nm. A few nanoparticles were distributed in the CNFs. Figure 6b shows the tip of the CNFs. The diameter of the CNFs still decreased gradually along the growth direction and finally formed a sharp cone. The diameter of the CNFs at the tip was 40 nm, and the angle of the sharp cone was about 6. Figure 6c shows the nanoparticle in the CNFs. The morphology of the nanoparticle was circular with a diameter of 65–70 nm. The EDS of the nanoparticle contained Cu and C elements, which further indicated that the catalyst was the Cu nanoparticles. The HR-TEM images of CNFs are shown in Figure 6d. The CNFs obtained at 500 °C were still disordered, and the selected area electron diffraction (SAED) patterns of CNFs were composed of three concentric circles corresponding to the (002), (100), and (110) planes of graphite [25].

### 3.4. Pore Structure of Phenolic Resin Pyrolytic Carbons

**Figure 7:**
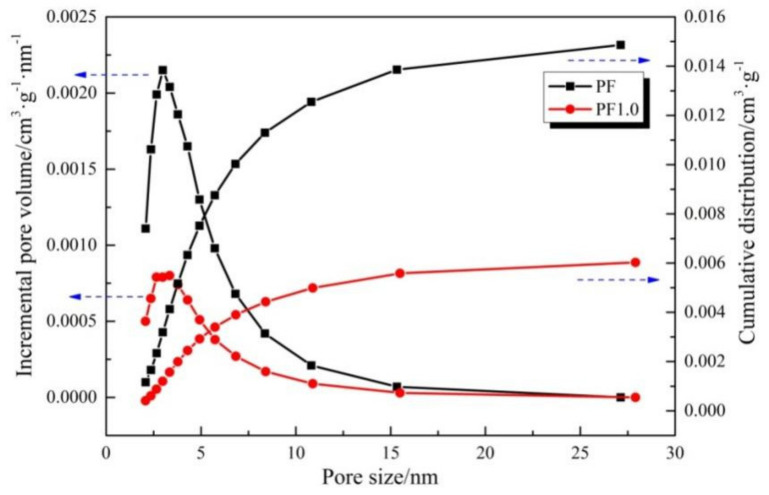
The pore size distribution and cumulative distribution curves of samples PF and PF1.0 heated at 500 °C for 3 h (The curve corresponding to the blue arrow pointing left represents the incremental pore volume of the samples, while the curve corresponding to the blue arrow pointing right represents the cumulative of the samples).

The BET method was used to calculate the specific surface area of phenolic resin pyrolytic carbons, and the BJH method was used to calculate the pore size distribution and pore volume of phenolic resin pyrolytic carbons [26]. Figure 7 shows the pore size distribution and cumulative distribution curves of PF and PF1.0 heated at 500 °C for 3 h.

The specific surface area of PF was 5.86591 m^2^/g, and that of PF1.0 was 1.76545 m^2^/g. The pore size distribution curve of the PF and PF1.0 pyrolysis products ranged from 2 to 30 nm, and the pore volume of sample PF1.0 with a pore diameter of 2–10 nm was significantly smaller than that of the sample PF. The cumulative distribution curves show that the total pore volume of PF1.0 was 0.0063 cm^3^/g, which was less than 0.01486 cm^3^/g of PF. The results show that the in situ synthesis of CNFs catalyzed by Cu could fill the pores and significantly reduce the pores’ volume of phenolic resin pyrolytic carbons.

### 3.5. Oxidation Resistance of Phenolic Resin Pyrolytic Carbons

In order to investigate the oxidation resistance of the pyrolysates of PF and PF1.0 as-calcined at 800 °C for 3 h, the TG–DSC testing was carried out in air heated at a rate of 10 °C/min from 30 °C to 1000 °C.

**Figure 8:**
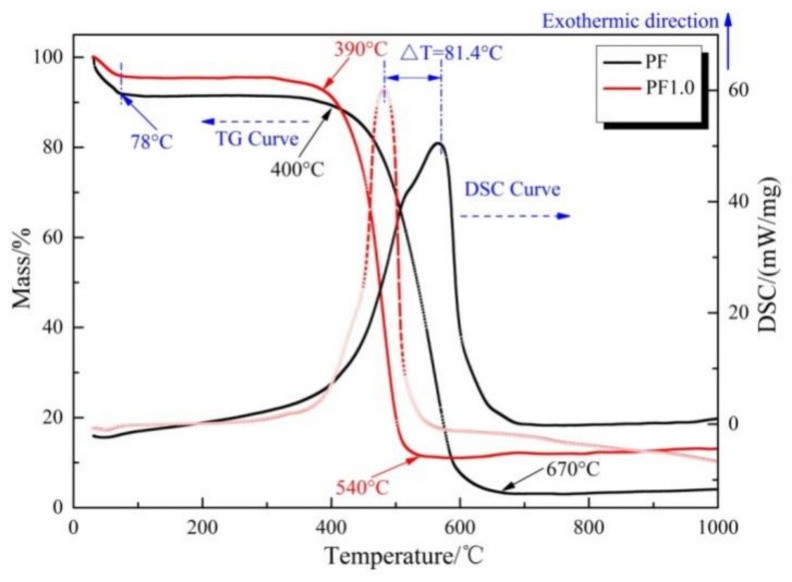
TG–DSC curves of samples PF and PF1.0 as–calcined at 800 °C for 3 h.

As shown in Figure 8, the TG data show that the weight loss of samples PF and PF1.0 could be divided into two stages.

The first stage of weight loss was 30–78 °C: the adsorbed water in the phenolic resin pyrolytic carbons was separated during the heating process. The weight loss rate of PF1.0 was lower than that of PF because the Cu-catalyzed CNFs reduced the pore volume of the phenolic resin pyrolytic carbons, thus reducing the amount of adsorbed water. The second stage of rapid oxidative weight loss for PF was 400–670 °C. The initial and final temperatures of sample PF1.0 were 390 °C and 540 °C, respectively. The maximum exothermic peak value of sample PF1.0 in the DSC curves was earlier than that of sample PF. The existence of Cu nanoparticles may promote the oxidation weight loss of phenolic resin pyrolytic carbons.

**Figure 9:**
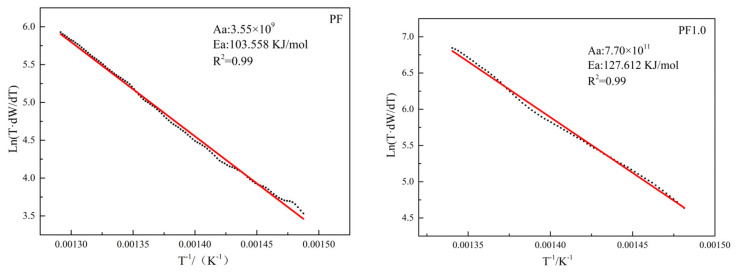
The oxidation reaction kinetics curves of samples PF and PF1.0.

The oxidation activation energies of PF and PF1.0 are shown in Figure 9.

The oxidation activation energy of PF1.0 and PF was 127.612 kJ/mol and 103.558 kJ/mol, respectively. The oxidation activation energy of PF1.0 was higher than that of PF, indicating that the addition of copper salts significantly improved the oxidation resistance of phenolic resin pyrolytic carbons [27].

### 3.6. Growth Mechanism of CNFs

**Figure 10:**
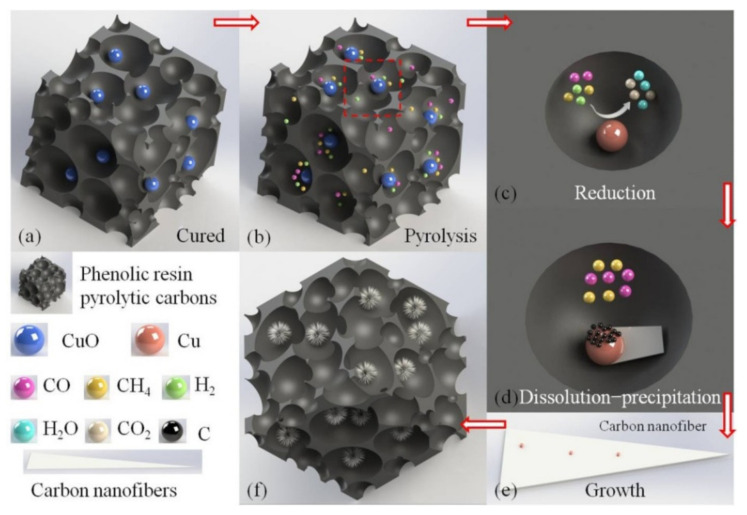
Growth mechanism of carbon nanofibers catalyzed by copper: (**a**) curing process of phenolic resin; (**b**) pyrolysis process of phenolic resin; (**c**) the reduction process of nanocopper particles; (**d**) the dissolution–precipitation process of carbon nanofiber; (**e**) the growth process of carbon nanofiber; (**f**) the structure of the final sample. (The direction of the arrows in figure indicates the progression of the growth process of carbon nanofibers catalyzed by nano-copper).

Figure 10 shows the growth mechanism of Cu-catalyzed carbon nanofibers. Ether bonds were generated upon curing of the phenolic resin via a dehydration condensation reaction of phenolic hydroxyl. CuO was formed by thermal decomposition of the copper nitrate [17,21], as shown in Figure 10a.

The combined action endows the cured sample with well-developed pores. During pyrolysis, the methylene bonds and ether bonds of phenolic resin break to form small molecular gases such as CH_4_, H_2_, and CO. These aggregated into the pores of cured phenolic resin [28,29], as shown in Figure 10b. CuO could be reduced to Cu via reaction, as can be seen in Equations (3)–(5) [21,30]; the thermal decomposition of phenolic resin would continue to generate the molecule gases. Here, the pores formed in the curing stage acted as the “reaction chamber”, as shown in Figure 10c.

4CuO(s) + CH_4_(g) → 4Cu(s) + CO_2_(g) + 2H_2_O(g) (3)

CuO(s) + CO(g) → Cu(s) + CO_2_(g) (4)

CuO(s) + H_2_(g) → Cu(s) + H_2_O(g) (5)

The diameter of reduced Cu particles was in the range of tens of nanometers. The reduced Cu nanoparticles crystallized well on the inside, but the conditions around the surface atoms and the atoms were different. The surface atoms of Cu nanoparticles were unstable, and the structural rearrangement of the surface atoms minimizes the energy of the system [17,21,30]. In turn, the melting temperature of Cu nanoparticles was lower than their normal temperature. Zhang et al. found that a smaller size of Cu nanoparticles endowed the Cu nanoparticles with a lower melting point [31].

The Cu nanoparticles melted into liquid droplets and had catalytic activity. Hydrocarbon gases such as CO and CH_4_ formed during the pyrolysis of phenolic resin adsorbed on the surface of liquid Cu nanoparticles and were catalytically cracked into active C atoms [32]. Because Cu atoms have a low solubility to C atoms [33], the active C atoms dissolved on one crystal plane and reached a supersaturated concentration; the active C atoms then diffused from the dissolved crystal plane to another specific crystal plane under the driving force of a concentration gradient [34]. They then precipitated into a “carbon film” with a flake structure on the specific crystal plane of Cu, as shown in Figure 10d. The Cu nanoparticles adsorbed CO and CH_4_ hydrocarbons continuously on different crystal planes and catalyzed the cracking of active C atoms. The active C atoms dispersed continuously on specific crystal planes and reached a supersaturated concentration. The carbon film with a flake structure developed into a CNF structure via dissolution–precipitation. The concentration of active C atoms decreased away from the Cu nanoparticles, and the tip diameter of the CNFs gradually decreased and finally formed CNFs with a tapered structure [35], as shown in Figure 10e. The catalytic activity of different Cu nanoparticles in different crystal planes was different due to the anisotropy of the Cu nanoparticles’ crystal planes. Meanwhile, the catalysis of the Cu nanoparticles was anisotropic and inhomogeneous due to the exposed crystal face and geometry [36]. Finally, the tapered carbon nanofibers were stacked in clusters and covered the surface of the phenolic resin pyrolytic carbons [37], as shown in Figure 10f.

## 4. Conclusions

The thermal decomposition of copper nitrate led to the formation of CuO. Then, the CuO was reduced to Cu nanoparticles by reducing the gas generated from the thermal decomposition of phenolic resin. Carbon nanofibers with a tapered structure were synthesized via Cu catalysis of the pyrolysis gas from phenolic resin at 500–800 °C. The synthesis and growth of CNFs were affected by the heating temperature and catalyst content. Increasing the pyrolysis temperature could promote the growth and development of carbon nanofibers but reduce the amount of CNFs. Increasing the amount of catalyst could increase the CNF content, but too much catalyst addition would affect the dispersion of Cu nanoparticles and catalytic activity. The introduction of copper salts increased the graphitization degree of phenolic resin pyrolytic carbons and reduced the pore volume of phenolic resin pyrolytic carbons. The combined action improved the oxidation resistance of phenolic resin pyrolytic carbons.

## Data Availability

The original contributions presented in the study are included in the article, further inquiries can be directed to the corresponding authors.

## References

[1] Yang Z.H., Liu B., Zhao H.C., Li J.F., Guo X.H., Zhang D.Q., Liu Z.H. (2023). Pyrolysis mechanism of composite binder composed of coal tar pitch and phenolic resin for carbon materials. J. Anal. Appl. Pyrol.

[2] Zanjanijam A.R., Wang X.Y., Ramezani M., Holberg S., Johnson P.A. (2023). Phenolic resin/coal char composites: Curing kinetics and thermal/ mechanical performance. Polymer.

[3] Yao J.Y., Wang J., Zhang Z.Z., Chen F., Liu Y.H. (2023). Ferrocenecarboxaldehyde modified terephthalaldehyde-phenolic resin: A comprehensive study on the synthesis, curing behavior, and pyrolysis process. Polymer.

[4] Gallegos I., Kemppainen J., Gissinger J.R., Kowalik M., Duin A.V., Wise K.E., Gowtham S., Odegard G.M. (2023). Establishing Physical and Chemical Mechanisms of Polymerization and Pyrolysis of Phenolic Resins for Carbon-Carbon Composites. Carbon Trends.

[5] Xing X.L., Niu X.R., Liu Y., Yang C.H., Wang S.J., Li Y., Jing X.L. (2021). In-depth understanding on the early stage of phenolic resin thermal pyrolysis through ReaxFF-molecular dynamics simulation. Polym. Degrad. Stabil..

[6] Rastegar H., Bavand-vandchali M., Nemati A., Golestani-Fard F. (2019). Phase and microstructural evolution of low carbon MgO-C refractories with addition of Fe-catalyzed phenolic resin. Ceram. Int..

[7] Niu J.W., Wang Z.F., Liu H., Ma Y., Pang H.X., Wang X.T. (2023). Response surface optimization of pitch phase change densification using composite phenolic resin co-carbonization to prepare high performance carbon refractories. J. Anal. Appl. Pyrol..

[8] Badaczewski F., Loeh M.O., Pfaff T., Dobrotka S., Wallacher D., Clemens D., Metz J., Smarsly B.M. (2019). Peering into the structural evolution of glass-like carbons derived from phenolic resin by combining small-angle neutron scattering with an advanced evaluation method for wide-angle X-ray scattering. Carbon.

[9] Zhao H.F., Xie D.D., Zhang S., Du F.L. (2020). Study on improving the high-temperature oxidation resistance of pyrolytic carbons of phenolic resin binder by in-situ formation of carbon nanotubes. React. Funct. Polym..

[10] Wei G.P., Zhu B.Q., Li X.C., Ma Z. (2015). Microstructure and mechanical properties of low-carbon MgO–C refractories bonded by an Fe nanosheet-modified phenol resin. Ceram. Int..

[11] Darban S., Kakroudi M.G., Vandchali M.B., Vafa N.P., Rezaei F., Charkhesht V. (2020). Characterization of Ni-doped pyrolyzed phenolic resin and its addition to the Al_2_O_3_-C refractories. Ceram. Int..

[12] Hu Q.H., Wang X.T., Wang Z.F. (2013). Preparation of graphitic carbon nanofibres by in situ catalytic graphitisation of phenolic resins. Ceram. Int..

[13] Wang J.K., Deng X.G., Zhang H.J., Zhang Y.Z., Duan H.J. (2017). Synthesis of carbon nanotubes via Fe-catalyzed pyrolysis of phenolic resin. Phys. E.

[14] Acauan L.H., Kaiser A.L., Wardle B.L. (2021). Direct synthesis of carbon nanomaterials via surface activation of bulk copper. Carbon.

[15] Bhaduri B. (2021). Synthesis of Cu catalyzed chemical vapor deposition grown Cu-CNFs on less porous graphite powder. Mater. Lett..

[16] Jiang H.T., Wang Y.X., Wang C.J., Li M.F., Xu Z.H. (2023). Catalyst optimization and reduction condition of continuous growth of carbon nanotubes on carbon fiber surface. Ceram. Int..

[17] Shaikjee A., Coville N.J. (2011). The effect of copper catalyst reducibility on low temperature carbon fibersynthesis. Mater. Chem. Phys..

[18] Tang K.H., Zhang A.L., Ge T.J., Liu X.F., Tang X., Li Y.J. (2021). Research progress on modification of phenolic resin. Mater. Today. Commun..

[19] Kibria M.A., Sripada P., Bhattacharya S. (2019). Rational design of thermogravimetric experiments to determine intrinsic char gasification kinetics. Proc. Combust. Inst..

[20] Gao Z.F., Zheng M.D., Zhang D.L., Zhang W.C. (2016). Low temperature pyrolysis propertiesand kinetics of non-coking coalin Chinese western coals. J. Energy Inst..

[21] Yu L.Y., Sui L.N., Qin Y. (2008). Low-temperature synthesis of carbon nanofibers by decomposition of acetylene with a catalyst derived from cupric nitrate. Chem. Eng. J..

[22] Rastegar H., Bavand-Vandchali M., Nemati A., Golestani-Fard F. (2018). Catalytic graphitization behavior of phenolic resins by addition of in situformed nano-Fe particles. Phys. E Low Dimens. Syst. Nanostruct..

[23] Anoshkin I.V., Nefedova I.I., Lioubtchenko D.V., Raisanen V.A. (2017). Single walled carbon nanotube quantification method employing the Raman signal intensity. Carbon.

[24] Chaunchaiyakui S., Yano T., Khoklang K., Krukowski P., Akai-Kasaya M., Saito A., Kuwahara Y. (2016). Nanoscale analysis of multiwalled carbon nanotube by tip-enhanced Raman spectroscopy. Carbon.

[25] Zheng R.T., Zhao Y., Liu H.P. (2006). Preparation, characterization and growth mechanismof platelet carbon nanofibers. Carbon.

[26] Beda A., Taberna P.L., Simon P., Ghimbeu C.M. (2018). Hard carbons derived from green phenolic resins for Na-ion batteries. Carbon.

[27] Liao N., Li Y.W., Shan J.B., Zhu T.B., Sang S.B., Jia D. (2018). Improved oxidation resistance of expanded graphite through nano SiC coating. Ceram. Int..

[28] Sobera M., Hetper J. (2003). Pyrolysis–gas chromatography—Mass spectrometry of cured phenolic resins. J. Chromatogr. A..

[29] Ouchi K. (1966). Infra-red study of structural changes during the pyrolysis of a phenol-formaldehyde resin. Carbon.

[30] Zhang Q., Wu X.F., Zhang Q.Q., Dong H.Z., Yu J.H., Dong L.F. (2018). Synthesis of multilayered carbon fiber arrays and their growth mechanism. Mater. Lett..

[31] Zhang Q., Yu L.Y., Cui Z.L. (2008). Effects of the size of nano-copper catalysts and reaction temperature on the morphology of carbon fibers. Mater. Res. Bull..

[32] Ren X., Zhang H., Cui Z.L. (2007). Acetylene decomposition to helical carbon nanofibers over supported copper catalysts. Mater. Res. Bull..

[33] Lopez G.A., Mittemeijer E.J. (2004). The solubility of C in solid Cu. Scr. Mater..

[34] Zhang Q., Du F.L., Dong L.F., Hao C.C. (2011). Formation of carbon fiber florets using copper tartrate catalyst precursors. Mater. Lett..

[35] Li X.J., Hao C.C., Lei Q.Q. (2012). Growth of carbon nanofibers catalyzed by silica-coated copper nanoparticles. Mater. Res. Bull..

[36] Qin Q., Zhang Q., Cui Z.L. (2004). Effect of synthesis method of nanocopper catalysts on the morphologiesof carbon nanofibers prepared by catalytic decomposition of acetylene. J. Catal..

[37] Kicinski W., Dyjak S. (2020). Transition metal impurities in carbon-based materials: Pitfalls, artifacts and deleterious effects. Carbon.

